# Population Genomic Assessment of Genetic Diversity and Structure in Mackerel Scad (*Decapterus macarellus*) in the South China Sea Based on RAD-seq

**DOI:** 10.3390/biology15191697

**Published:** 2026-09-23

**Authors:** Yi Li, Shuai Zhang, Min Li, Jiangtao Fan, Yancong Cai, Yutao Yang, Shannan Xu, Zuozhi Chen

**Affiliations:** 1 South China Sea Fisheries Research Institute, Chinese Academy of Fishery Sciences, Guangzhou 510300, China; 2 College of Fisheries and Life Sciences, Shanghai Ocean University, Shanghai 201306, China; 3 Key Laboratory for Sustainable Utilization of Open-Sea Fishery, Ministry of Agriculture and Rural Affairs, Guangzhou 510300, China; 4 Guangdong Provincial Observation and Research Station for Ecosystem in the Pearl River Estuary, Guangzhou 510300, China

**Keywords:** *Decapterus macarellus*, RAD-seq, population structure, genetic diversity, South China Sea

## Abstract

The mackerel scad (*Decapterus macarellus*) is an important pelagic fish in the South China Sea (SCS), where it supports light purse-seine fisheries. Genome-wide information on its genetic diversity and population structure remains limited, although it is needed for stock identification and sustainable management. Restriction-site-associated DNA sequencing (RAD-seq) was used to analyze 112 *D. macarellus* individuals collected from Xisha, Dongsha, and Nansha Islands in the SCS, with Dongsha and Nansha sampled in April 2025 and Xisha sampled in September 2025; the congener *Decapterus macrosoma* was included as an outgroup for analyses requiring an external reference. A total of 12,080 high-quality single-nucleotide polymorphisms (SNPs) across 1087 retained loci were used for within-species analyses. The three sampling groups showed moderate-to-high genetic diversity (π = 0.02045–0.03191; *H*_O_ = 0.01669–0.02497). Pairwise F_ST_ values ranged from 0.0086 to 0.0120 and D_XY_ values ranged from 0.00156 to 0.00174, while principal component analysis, ADMIXTURE, and phylogenetic analyses indicated low genome-wide differentiation among the sampled regions. The TreeMix results were treated as exploratory and showed close allele–frequency relationships among the sampling groups. Genotype–environment association analysis identified putative association signals across the environmental variables examined, with chlorophyll-a, salinity, and nitrate yielding the largest numbers of candidate loci. Overall, these results indicate low neutral genetic differentiation among the sampled groups. Because sampling location and month were confounded and the data are based primarily on neutral genomic markers, the present results should be considered a genomic baseline rather than definitive evidence for a single fishery management unit.

## Simple Summary

Mackerel scad is an important pelagic fish supporting purse-seine fisheries in the South China Sea, but its population genomic structure remains poorly understood. We analyzed genome-wide genetic variation in 112 fish from the Xisha, Dongsha, and Nansha Islands using restriction-site-associated DNA sequencing. Dongsha and Nansha were sampled in April 2025, whereas Xisha was sampled in September 2025. The three sampling groups retained moderate-to-high genetic diversity and showed low genetic differentiation. Analyses of ancestry, principal components, phylogenetic relationships, and historical relationships consistently indicated extensive genetic similarity across the surveyed regions. We also detected putative genotype–environment associations, with chlorophyll-a, salinity, and nitrate yielding the largest numbers of candidate loci; these results are exploratory and require further validation. The results provide the first genome-scale baseline for mackerel scad in the South China Sea. Because sampling month was confounded with location and the inference is based mainly on neutral genomic markers, definitive fishery management-unit delineation requires broader, seasonally matched and temporally replicated sampling.

## 1. Introduction

The mackerel scad (*Decapterus macarellus*) is a pelagic fish of the family Carangidae and is widely distributed in tropical and subtropical waters. In the South China Sea (SCS), this species is one of the major targets of light purse-seine fisheries and represents an important offshore pelagic fishery resource. Historical acoustic surveys estimated its biomass at 303,800 tonnes in the central–southern SCS in spring 2013 and 161,900 tonnes in the northern offshore SCS in spring 2015 [1,2]. Its commercial importance and broad distribution highlight the need for genetic information that can support stock identification and sustainable management.

Population genetic information is useful for fisheries assessment because it can reveal patterns of genetic differentiation that are not always reflected by geographic distance or administrative boundaries. In marine fishes, genetic differentiation is often weak because large effective population sizes, pelagic eggs and larvae, adult dispersal, and the absence of strong physical barriers can promote genetic exchange among regions [3,4]. However, population structure may still arise when oceanographic processes, spawning behavior, ecological gradients, or historical isolation reduce genetic exchange. Previous studies on commercially important fishes have shown that molecular markers can inform stock identification and fishery management [5,6,7]. Genome-wide genetic data are therefore needed to characterize population structure and genetic differentiation in *D. macarellus* across the SCS.

Previous studies on *D. macarellus* have mainly focused on biological characteristics [8,9,10,11], stock assessment [1,2], habitat distribution [12,13], taxonomic clarification [14], and egg identification [15]. Genetic information remains limited, with available studies mainly involving mitochondrial genome sequencing [16], mtDNA-based population analyses [14], or RFLP markers [17]. Although these studies provided useful preliminary information, single-locus or low-throughput markers have limited power to detect subtle genetic differentiation in widely dispersing marine fishes. A genome-wide SNP dataset is therefore needed to assess genetic diversity and population structure in *D. macarellus* more robustly.

Restriction-site-associated DNA sequencing (RAD-seq) provides an efficient approach for generating genome-wide single-nucleotide polymorphisms (SNPs), especially for non-model species without a high-quality reference genome [18]. Compared with traditional molecular markers, RAD-seq can improve the resolution of population genetic analyses and has been increasingly used to assess genetic diversity and differentiation in aquatic resources [19]. Genome-wide SNPs can also be used to identify candidate loci associated with environmental gradients, providing preliminary information on environment-associated genetic variation that may be useful for long-term genetic monitoring.

In this study, we used RAD-seq to analyze 112 *D. macarellus* individuals collected from three geographic regions of the SCS: Xisha, Dongsha, and Nansha Islands. The objectives were to (1) estimate genome-wide genetic diversity in the sampled groups; (2) evaluate genetic differentiation and population structure among the three geographic sampling groups; (3) explore putative SNP–environment associations; and (4) provide baseline genomic information relevant to future fishery management-unit delineation and the sustainable exploitation of *D. macarellus* in the SCS.

## 2. Materials and Methods

### 2.1. Collection and Processing of D. macarellus Samples

Samples of *D. macarellus* were collected from the South China Sea in April and September 2025 using the research vessel Zhong Yuke 301, with a standardized light purse-seine method. Dongsha (DS) and Nansha (NS) samples were collected in April 2025, whereas Xisha (XS) samples were collected in September 2025. At each sampling station, a 60 min operation was conducted, during which stable lighting was maintained to attract fish schools. Because sampling month was confounded with location, population genetic results were interpreted as comparisons among geographic sampling groups rather than as a strictly season-controlled spatial design. Accordingly, spatial and temporal effects could not be independently estimated under the current sampling design. After landing, catches were sorted and identified to species level based on morphological characteristics according to the Synopsis of Chinese Fishes [20]. *D. macarellus* and its congener *Decapterus macrosoma* were separated carefully. *D. macrosoma* was used as an outgroup only in analyses requiring an external reference and was not included in within-species estimates of genetic diversity or population structure for *D. macarellus*. All individuals were measured for body length, body weight, and other biological information and were immediately frozen for transport to the laboratory. From each sampling area, muscle tissue from 35 to 42 individuals was taken, preserved in anhydrous ethanol, and stored at −20 °C for subsequent DNA extraction. Sampling locations and sample sizes are shown in Figure 1.

### 2.2. Genomic DNA Extraction and RAD-seq

Total genomic DNA was extracted using a StarSpin Universal DNA Kit (GenStar, Beijing, China) following the manufacturer’s protocols. DNA integrity was assessed by 1.2% agarose gel electrophoresis, and DNA concentration was measured using a Qubit 3.0 fluorometric system (Thermo Fisher Scientific, Waltham, MA, USA). Samples with clear electrophoretic bands and no obvious signs of severe degradation were considered suitable for library preparation. Samples were normalized to 20 ng/µL, and 200 ng of genomic DNA per sample was used for library preparation.

For RAD-seq, genomic DNA was double-digested with EcoRI-HF and MseI (New England Biolabs, Ipswich, MA, USA) in a 20 μL reaction. The mixture was incubated at 37 °C for 8 h, followed by heat inactivation at 65 °C for 20 min. Subsequently, EcoRI adapters containing sample-specific barcodes and universal MseI adapters were ligated to the digested fragments using T4 DNA ligase (New England Biolabs, Ipswich, MA, USA) at 16 °C for 8 h, followed by heat inactivation at 65 °C for 20 min. Barcoded products were pooled in equal amounts, size-selected for fragments of 400–550 bp, purified, and PCR-amplified. Library quality and fragment size distribution were assessed prior to sequencing. Libraries were sequenced on a NovaSeq 6000 platform (Illumina, Inc., San Diego, CA, USA) using 150 bp paired-end reads (PE150), generating approximately 1.0 Gb of raw data per sample.

### 2.3. Data Processing and Analysis

#### 2.3.1. SNP Calling

Raw reads were processed using the process_radtags module in Stacks v2.68 [21] for demultiplexing and quality control using the EcoRI–MseI enzyme pair. Reads with unrecognized barcodes or ambiguous cut sites were removed (-c), low-quality reads were filtered (-q), and single mismatches in barcodes and restriction sites were rescued (-r). Reads shorter than 140 bp were discarded (--len_limit 140), and all reads were truncated to 135 bp (-t 135). Because a reference genome was unavailable for *D. macarellus*, clean reads were aligned to the genome of the congener *Decapterus maruadsi* (NCBI accession: GCA_030347415.2) using BWA-MEM v0.7.17-r1188 [22] with default settings. After that, SAMtools v1.18 [23] was used to convert the alignment files to BAM format. Alignment quality was summarized from the gstacks BAM statistics, including unmapped records and primary alignments retained after mapping-quality and soft-clipping filters. SNPs were called with gstacks in Stacks v2.68 using default settings, followed by population-level filtering with populations. For the within-species dataset (XS, DS, and NS), loci were required to occur in all three groups (-p 3) and to be genotyped in ≥80% of individuals within each group (-r 0.8). For the dataset including the HND outgroup, the corresponding criteria were -p 4 and -r 0.8. Thus, retained loci allowed no more than 20% missing genotypes within any sampling group. No additional MAF filter was applied during the populations step (MAF cutoff = 0). Filtered SNPs were exported in VCF format together with population genetic statistics.

#### 2.3.2. Population Genetic Analysis

Genetic diversity was assessed using the populations module in Stacks v2.68, including the proportion of polymorphic loci, nucleotide diversity (π), observed heterozygosity (*H*_O_), expected heterozygosity (*H*_E_), minor allele frequency (MAF), genetic divergence (D_XY_), and pairwise fixation indices (F_ST_).

#### 2.3.3. Population Stratification and Genetic Structure

To elucidate the genetic structure of *D. macarellus* groups, multiple analytical approaches were employed. First, principal component analysis (PCA) was performed using PLINK v1.9.0 [24] based on the VCF file. After calculating the principal components, scatter plots of individual PC scores were generated in R v4.3.3 [25] to visualize the distribution of individuals along the principal components. Additionally, Bayesian clustering analysis was conducted using ADMIXTURE v1.3.0 [26], with the number of ancestral populations (*K*) ranging from 1 to 6 and five cross-validation runs for each *K* value to determine the optimal number of ancestral populations. The results were visualized using R v4.3.3 [25] to generate line plots of cross-validation errors and population structure plots. Furthermore, a maximum likelihood method was used to construct a phylogenetic tree. First, a custom Python 3 script was used to convert the VCF file into an sPHYLIP alignment format. Then, FastTree v2.1.11 [27] was run under the GTR model to generate a tree in N_e_wick format [28]. The resulting tree was uploaded to the iTOL online platform for visualization and annotation [29]. Finally, pairwise F_ST_ values among groups were calculated, and a heatmap was drawn in R to visualize the genetic differentiation matrix [30].

#### 2.3.4. TreeMix Analysis of Historical Relationships

To examine historical relationships among the geographic sampling groups of *D. macarellus*, TreeMix analysis was performed using the SNP dataset. For the TreeMix input dataset, PLINK v1.9.0 was used for LD pruning with a 50-SNP window, 5-SNP step, and an *r*^2^ threshold of 0.2. The pruned data were converted to PLINK transposed format, and allele frequencies were calculated using the --freq option. The resulting output was compressed and converted to TreeMix input format. Using HND as the outgroup, TreeMix v1.13 [31] was then used to fit models with one to three migration edges (*m* = 1–3) and examine relationships and allele–frequency covariance among sampling groups. Given the low overall differentiation and the limited number of sampled groups, migration-edge signals were interpreted as exploratory patterns rather than direct estimates of migration rates or contemporary demographic connectivity.

#### 2.3.5. Environmental Association Analysis

To explore associations between genetic variation and environmental factors in *D. macarellus*, a genome-wide genotype–environment association analysis was performed. Marine environmental variables were downloaded from the Bio-ORACLE database [32], including mean sea surface temperature, mean salinity, mean chlorophyll-a concentration, mean bathymetry, mean pH, dissolved oxygen, nitrate concentration, phosphate concentration, and zooplankton biomass. For temporally varying oceanographic variables, mean surface layers for 2010–2020 were used, whereas bathymetry was treated as a static topographic variable. Raster layers were downloaded in GeoTIFF format at a spatial resolution of 0.05°. Sampling coordinates were referenced to WGS84 (EPSG:4326), and site-specific environmental values were extracted from the corresponding raster cells using raster package v3.6-32 in R. Environmental variables were standardized in R v4.3.3, and the SNP dataset was converted to a genotype matrix for LFMM analysis.

LFMM was fitted using lfmm_ridge in the lfmm package v1.1 with *K* = 1. This value was selected because the ADMIXTURE cross-validation error was lowest at *K* = 1 and the PCA showed no clear discrete genetic clusters. SNP–environment associations were tested using lfmm_test, generating raw *p*-values and corrected *p*-values; *q*-values were additionally calculated from raw *p*-values using the Benjamini–Hochberg procedure. Candidate SNPs were defined using corrected *p* < 0.01, and Manhattan plots were generated from the corrected *p*-values. Given the limited number of sampling locations, the LFMM analysis was treated as exploratory, and candidate loci were interpreted as putative genotype–environment association signals rather than direct evidence of local adaptation.

## 3. Results

### 3.1. RAD-seq and SNP Identification

We performed RAD-seq on 112 *D. macarellus* individuals collected from the SCS, generating 818,749,182 raw reads (1,356,192–77,394,276 per individual). After quality filtering, 726,389,494 reads were retained (88.72%), including 341,603,528 properly paired read pairs. According to the gstacks BAM statistics, 631,038,460 BAM records were processed for the within-species dataset; 318,062,447 records (50.40%) were mapped (i.e., not flagged as unmapped); and 183,258,346 primary alignments (29.04% of all BAM records) were retained after mapping-quality and soft-clipping filters. Mean sequencing depth was 22.95× (range: 3.02×–96.72×). Before population-level filtering, the within-species Stacks dataset contained 210,778 catalog loci and 915,951 SNP sites. After applying -p 3 and -r 0.8, 1087 loci and 12,080 variant SNPs were retained, corresponding to 1.32% of the pre-filter SNP sites and an average of 11.1 variant SNPs per retained locus. The mean genotyped length per retained locus was 184.08 bp, and the final 112-individual VCF had an overall genotype missing rate of 8.83%. For analyses requiring an outgroup, the dataset including *D. macrosoma* contained 1064 loci and 15,090 variant SNPs after population-level filtering.

### 3.2. Genomic Diversity of D. macarellus

To assess genetic diversity among the three geographic sampling groups of *D. macarellus* in the SCS, we calculated several key genetic parameters for the three geographic sampling groups (Table 1). The values of *H*_O_, *H*_E_, MAF, and π ranged from 0.01669 to 0.02497, 0.02004 to 0.03144, 0.01061 to 0.01672, and 0.02045 to 0.03191, respectively. Among the three groups, XS showed the highest values for all four genetic diversity indices. For DS and NS, *H*_O_ was 0.01669 and 0.01712, *H*_E_ was 0.02004 and 0.02050, MAF was 0.01061 and 0.01068, and π was 0.02045 and 0.02089, respectively (Table 1).

### 3.3. Genetic Differentiation Levels and Genetic Structure of D. macarellus

F_ST_ and D_XY_ were calculated to assess the levels of genetic differentiation among the three geographic sampling groups of *D. macarellus* (Table 2). Pairwise F_ST_ values ranged from 0.0086 to 0.0120 (Figure 2), and D_XY_ values ranged from 0.00156 to 0.00174. ADMIXTURE analysis identified *K* = 1 as the best-supported model (Figure 3A), indicating no strong ancestry subdivision among the 112 *D. macarellus* individuals under this analysis. For *K* values ranging from 1 to 6, no clear geographic clustering was observed across individuals from XS, DS, and NS (Figure 3B). The first two principal components explained 18.6% and 6.02% of the variation, respectively. The PCA plot (Figure 4A) showed that all samples clustered closely together, with NS and DS largely overlapping and nearly indistinguishable, while XS was slightly separated from them along PC2. Because XS was sampled in September whereas DS and NS were sampled in April, this modest displacement cannot be attributed solely to geographic differentiation. The phylogenetic tree also showed that DS and NS individuals were largely interspersed, whereas XS individuals tended to form a relatively distinct branch (Figure 4B).

In summary, although a slight displacement of XS was observed in the PCA and phylogenetic tree, the low pairwise F_ST_ values and the ADMIXTURE result at *K* = 1 indicated limited genome-wide differentiation among the three sampling groups.

### 3.4. Historical Relationships Inferred by TreeMix

TreeMix analysis was used to examine historical relationships and migration-edge signals among the three geographic sampling groups of *D. macarellus*, with *D. macrosoma* (HND) as the outgroup and *m* = 1–3 migration edges. Across all models, HND was clearly separated from the three *D. macarellus* groups, whereas XS, DS, and NS clustered within the *D. macarellus* lineage (Figure 5). DS and NS were more closely related to each other than to XS. Migration-edge signals differed among models and reflected alternative patterns of allele–frequency covariance among sampling groups. These signals were interpreted as exploratory and not as direct estimates of migration rates or contemporary demographic connectivity.

### 3.5. Environmental Association Analysis of D. macarellus

We performed genotype–environment association analysis to examine potential associations between SNP variation and environmental factors in *D*. *macarellus*. Using a corrected *p*-value threshold of <0.01, candidate SNPs were identified for all nine environmental variables examined (Figure 6), with the number of associated loci varying among environmental factors.

Chlorophyll-a showed the largest number of candidate SNPs (174), followed by salinity (114), nitrate (108), pH (92), phosphate (87), sea surface temperature (63), dissolved oxygen (55), water depth (47), and zooplankton biomass (1). Chlorophyll-a, salinity, and nitrate yielded relatively higher numbers of candidate loci than the other environmental variables. Overall, candidate genotype–environment associations were detected across all nine environmental variables, with marked differences in the number of candidate loci among environmental factors.

## 4. Discussion

### 4.1. Population Genetic Diversity of D. macarellus

In this study, genome-wide SNPs obtained by RAD-seq were used to evaluate the genetic diversity of *D. macarellus* from three geographical regions of the SCS. The genetic diversity indices showed that all sampled groups maintained moderate-to-high levels of variation. This result is consistent with previous COI-based evidence showing high haplotype diversity in *D. macarellus* from the SCS and eastern Indian Ocean [14]. At the genomic level, our results indicate that this species still retains substantial genetic resources in the surveyed regions.

The maintenance of this level of genetic diversity may be related to several biological and ecological factors. First, a large effective population size (Ne) can reduce the effect of genetic drift and maintain genetic variation over time, although Ne was not estimated in this study. The relatively high biomass reported for *D*. *macarellus* in the SCS may be consistent with this possibility but should not be regarded as a direct estimate of Ne [1,2,33]. Second, pelagic eggs and larvae, schooling behavior, and adult dispersal may promote genetic exchange among regions [3,15,34]. Third, the relatively long spawning season reported for *D. macarellus* in different regions may increase opportunities for genetic exchange among cohorts [8,10,11]. These factors may help explain the observed genetic diversity, but they were not directly tested in the present study.

Similar patterns of relatively high genetic diversity have been observed in other exploited marine fishes, including copper redhorse (*Moxostoma hubbsi*), North Sea sole (*Solea solea*), and Atlantic cod (*Gadus morhua*) [35,36,37]. Nevertheless, genetic diversity may respond slowly to demographic changes, and genetic erosion may therefore become detectable only after a time lag. Thus, the relatively high genetic diversity observed here does not preclude future changes in genetic status. Continued genetic monitoring alongside conventional stock assessment would help detect such changes over time.

### 4.2. Genetic Structure of D. macarellus

The population structure analyses revealed low genetic differentiation among the three sampled regions of *D. macarellus* in the SCS. Pairwise F_ST_ values ranged from 0.0086 to 0.0120, and D_XY_ values ranged from 0.00156 to 0.00174. PCA, ADMIXTURE, and phylogenetic analyses also indicated weak genome-wide differentiation. Together, these neutral-marker results indicate limited genome-wide differentiation among the sampled groups, but they do not by themselves demonstrate contemporary demographic connectivity.

Several biological and historical processes could contribute to the weak genetic structure observed here. The prolonged spawning season and pelagic early life stages of *D. macarellus* may facilitate dispersal by currents [15], while adult schooling and seasonal movements may further enhance mixing among regions [8,10,11]. Similar weak differentiation has been reported in other widely distributed marine fishes, including *Larimichthys polyactis*, *Dissostichus mawsoni*, and *Etelis oculatus* [38,39,40]. Historical sea-level changes in the western Pacific marginal seas may also have influenced demographic history and post-glacial connectivity in regional marine fishes [41], and a large effective population size can reduce the rate of differentiation by weakening genetic drift [4,33]. These mechanisms remain plausible explanations rather than processes directly tested by the present dataset.

The slight separation of XS in the PCA and phylogenetic tree should be interpreted cautiously. This pattern may reflect minor genetic differences, temporal variation, or sampling effects. DS and NS were collected in April, whereas XS was collected in September; therefore, the observed XS pattern may include a temporal component related to differences in sampling season, potentially including seasonal movement or recruitment. Because sampling month and location were confounded, the XS displacement cannot be attributed solely to geographic population structure.

Overall, the results indicate low neutral genetic differentiation among the surveyed groups of *D. macarellus* in the SCS. From a management perspective, these data provide baseline information but are insufficient on their own to define a single fishery management unit. Because the current sampling covered only three regions and two sampling months, management-unit delineation should be supported by seasonally matched sampling and additional ecological or demographic evidence.

### 4.3. Candidate Environment-Associated Genetic Variation in D. macarellus

The LFMM analysis identified candidate SNPs associated with all nine environmental variables, although the number of candidate loci differed markedly among variables. Chlorophyll-a yielded the largest number of candidate SNPs, followed by salinity and nitrate, whereas zooplankton biomass was associated with only one candidate locus. Together, these patterns suggest that genomic variation in *D*. *macarellus* may covary with multiple components of the marine environment.

Chlorophyll-a, nitrate, and phosphate represent productivity-related environmental gradients, and comparable genotype–environment associations involving chlorophyll-a or nutrients have been reported in several marine fishes, including *Sardinella longiceps*, *Reinhardtius hippoglossoides*, and *Eleginops maclovinus* [42,43,44]. However, such relationships may vary among species and regions; in *Sardinella lemuru*, significant associations were more evident for sea surface temperature than for chlorophyll-a [45]. Salinity may also be biologically relevant through physiological responses involving osmoregulation, ion transport, and water balance [46,47]. Candidate associations with pH, sea surface temperature, dissolved oxygen, and water depth further suggest that genomic variation may covary with several physicochemical gradients. Depth-related biochemical and morphological variation has been documented in other fishes [48,49]. In contrast, the weak association with zooplankton biomass may partly reflect the strong spatial and temporal variability in biotic factors and their indirect effects through trophic interactions; both abiotic and biotic variables have been associated with genetic patterns in fishes [50].

Nevertheless, these associations require cautious interpretation. Only three geographic sampling locations were available, limiting independent environmental replication, and sampling month was confounded with location. For temporally varying variables, the Bio-ORACLE layers represented 2010–2020 decadal mean surface conditions rather than conditions measured contemporaneously with fish sampling in April and September 2025. The detected associations should therefore be interpreted as relationships with broad-scale, long-term environmental gradients rather than responses to environmental conditions at the time of sampling. Although *K* = 1 was supported by ADMIXTURE and the PCA, subtle residual population structure may remain. In addition, spatial autocorrelation and correlations among environmental predictors could generate non-independent association signals that cannot be robustly disentangled with only three sampling locations. Accordingly, the identified loci should be regarded as exploratory genotype–environment association signals rather than direct evidence of local adaptation. Broader spatial and temporal sampling, seasonally matched environmental observations, and functional validation will be required to confirm these patterns.

### 4.4. Limitations and Future Prospects

This study has several limitations. First, sampling was limited to three regions in the SCS and did not cover the full distribution of *D. macarellus*, limiting inference about population structure and genetic differentiation beyond the surveyed regions. Second, sampling month was confounded with location: DS and NS were collected in April, whereas XS was collected in September, so spatial and temporal effects cannot be separated for the slight XS displacement. Third, the absence of a chromosome-level annotated reference genome limited functional interpretation of candidate environment-associated SNPs. Finally, genotype–environment associations were constrained by broad-scale environmental layers and limited independent environmental replication. The temporally varying Bio-ORACLE layers represented 2010–2020 decadal mean conditions rather than contemporaneous 2025 sampling conditions, and potential correlations among environmental variables could not be robustly disentangled with only three sampling locations. These constraints reinforce the exploratory interpretation of the LFMM results.

Future studies should expand spatial coverage in the SCS and adjacent waters and incorporate seasonally matched, temporally replicated sampling. A chromosome-level reference genome and functional annotation of candidate loci would further improve interpretation of environment-associated signals. Repeated genomic surveys would help determine whether the genetic similarity observed among the sampled groups is stable through time and provide stronger support for adaptive fishery management of *D. macarellus*.

## 5. Conclusions

This study provides the first genome-scale assessment of genetic diversity and population structure of *Decapterus macarellus* in the South China Sea based on RAD-seq data. The Xisha, Dongsha, and Nansha sampling groups showed moderate-to-high genetic diversity and low neutral genetic differentiation (pairwise F_ST_ = 0.0086–0.0120). These findings provide a genomic baseline for future stock delineation; however, the temporally unbalanced sampling design and reliance mainly on neutral genomic markers do not justify defining the sampled groups as a single fishery management unit. Putative genotype–environment associations were detected across the environmental variables examined, with relatively more candidate loci associated with chlorophyll-a, salinity, and nitrate; these exploratory signals require broader spatial replication and independent validation. Further studies with broader spatial coverage, temporal replication, and functional validation are needed to support long-term genetic monitoring and sustainable management of this commercially important pelagic fish.

## Figures and Tables

**Figure 1:**
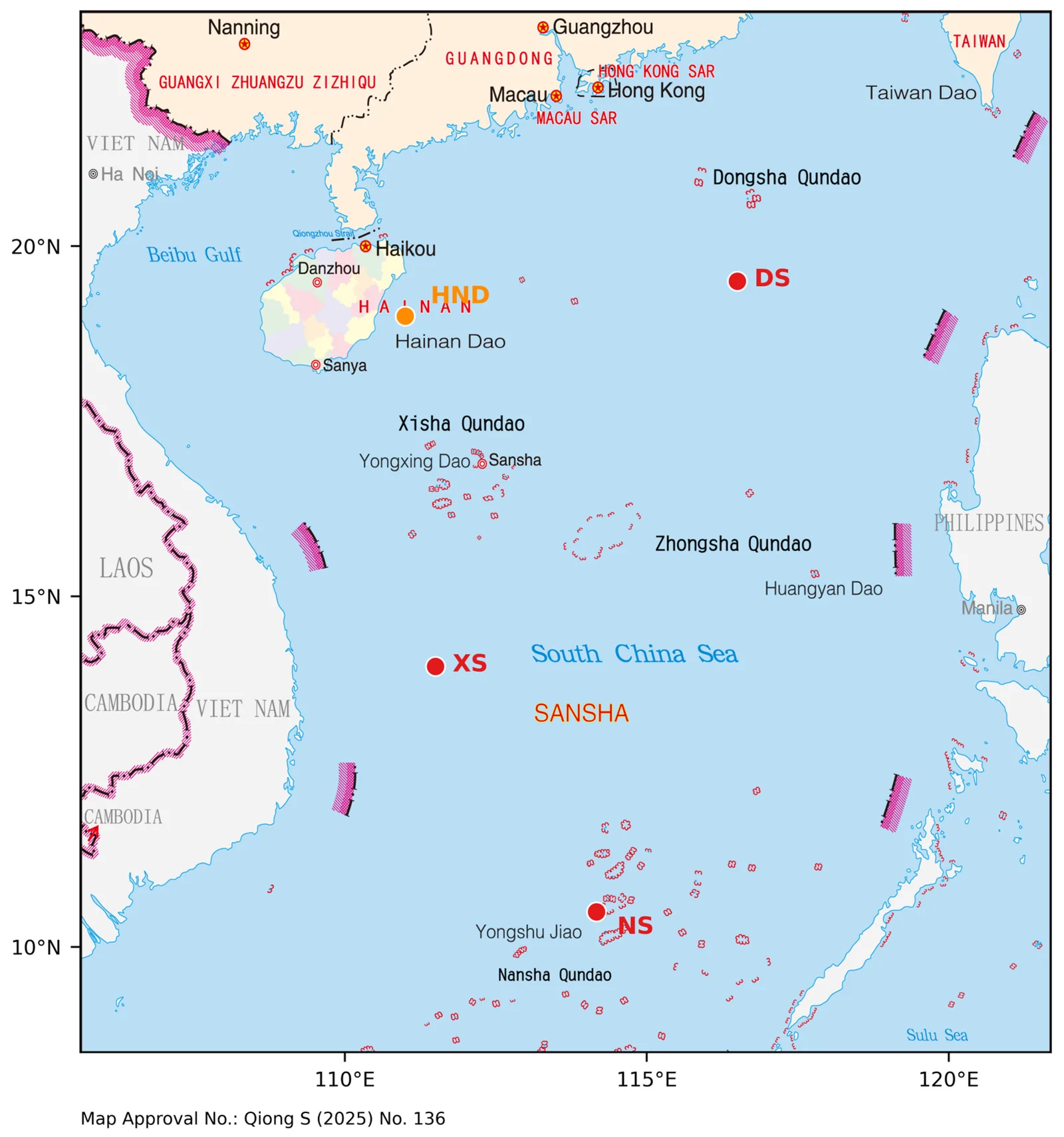
Sampling locations for the three geographic sampling groups of *D. macarellus* (XS: Xisha Islands, *n* = 42; NS: Nansha Islands, *n* = 35; DS: Dongsha Islands, *n* = 35) and for the outgroup *D. macrosoma* (HND: Hainan, *n* = 35).

**Figure 2:**
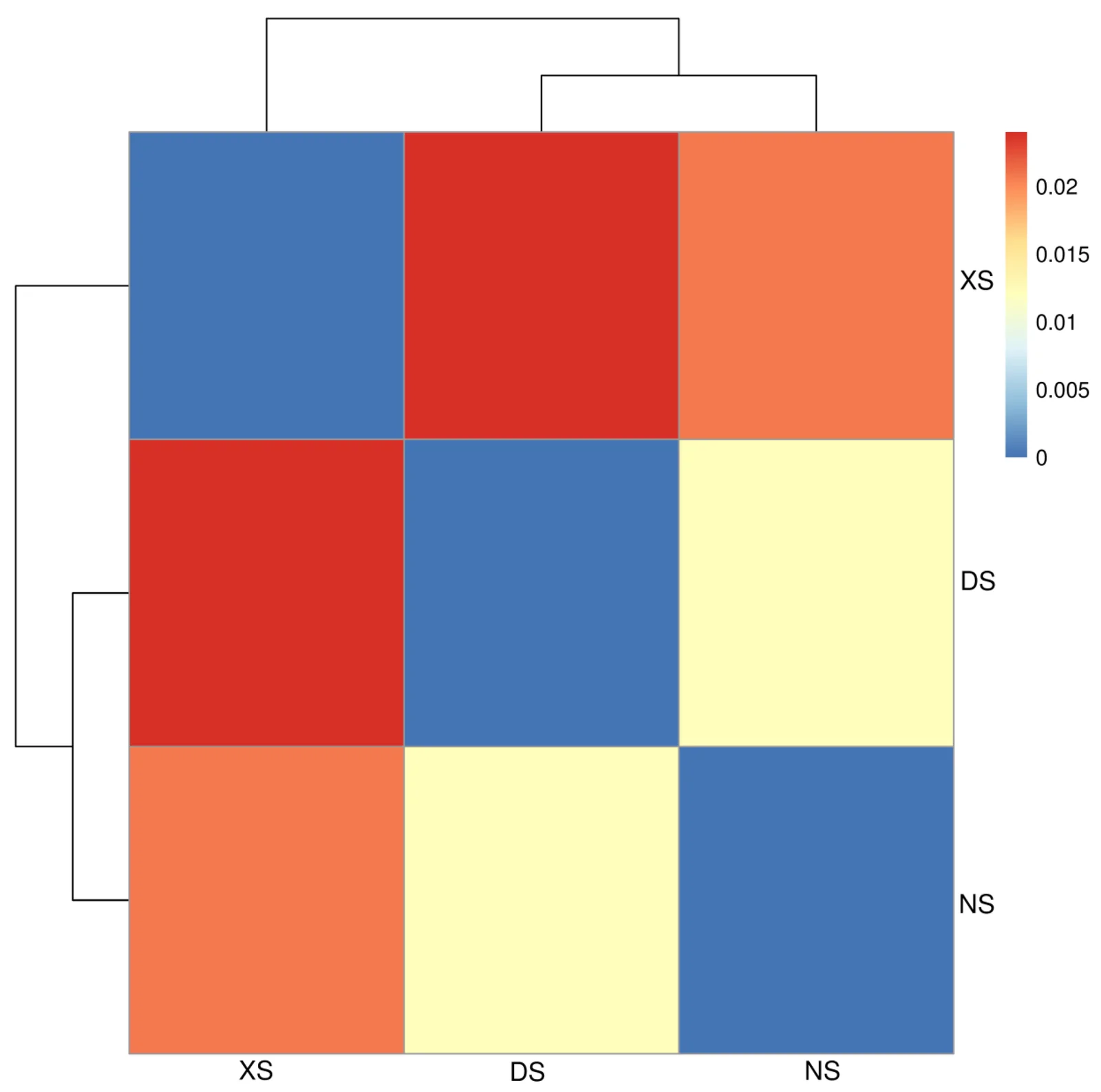
Pairwise fixation indices (F_ST_) heatmap of *D. macarellus* based on genomic SNP data.

**Figure 3:**
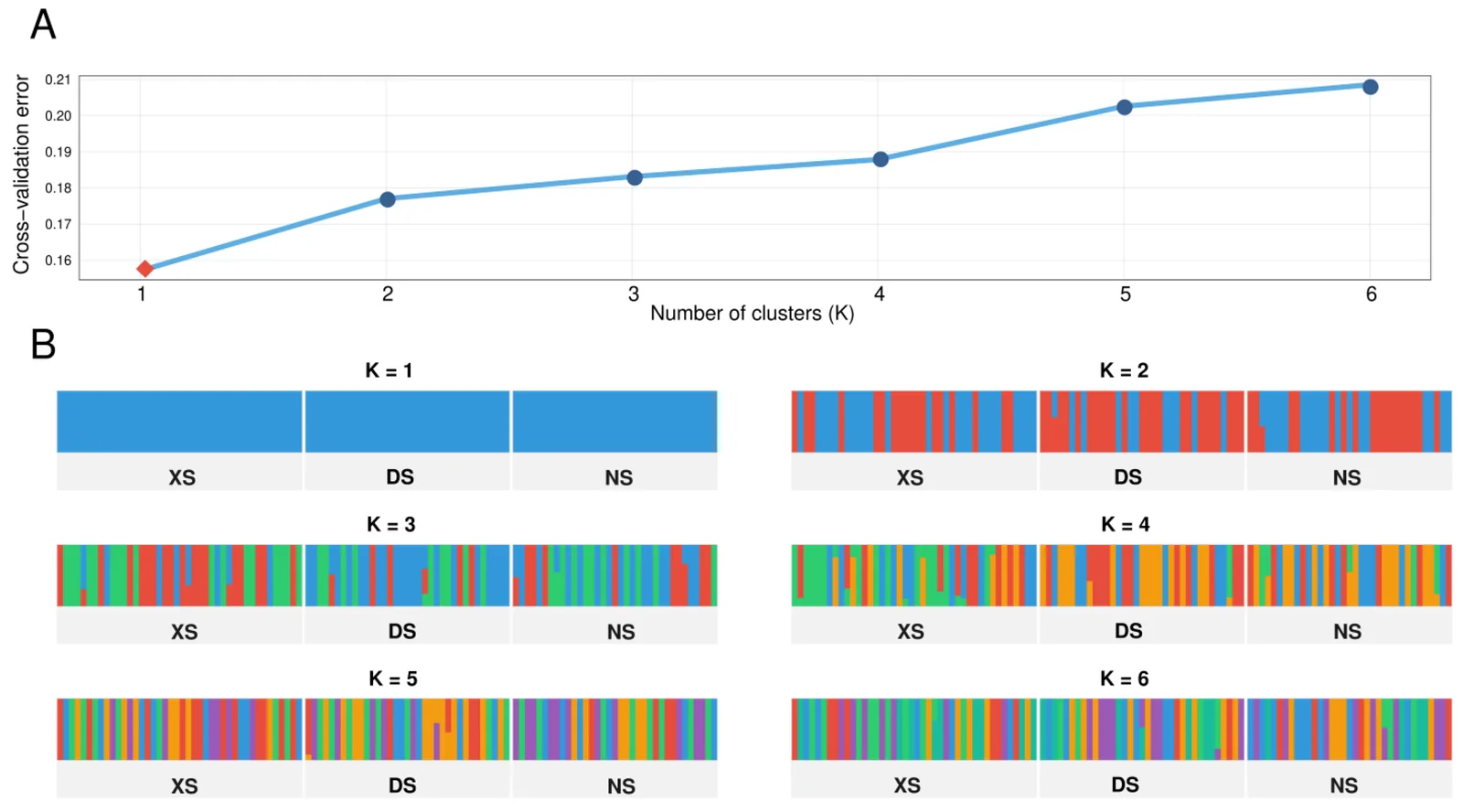
Population genetic structure analysis of *D. macarellus* based on genomic SNP data. (**A**) Cross-validation error plot used to determine the optimal number of ancestral populations (*K* value) in ADMIXTURE analysis; the minimum error point corresponds to *K* = 1. (**B**) Genetic ancestry component map based on ADMIXTURE analysis.

**Figure 4:**
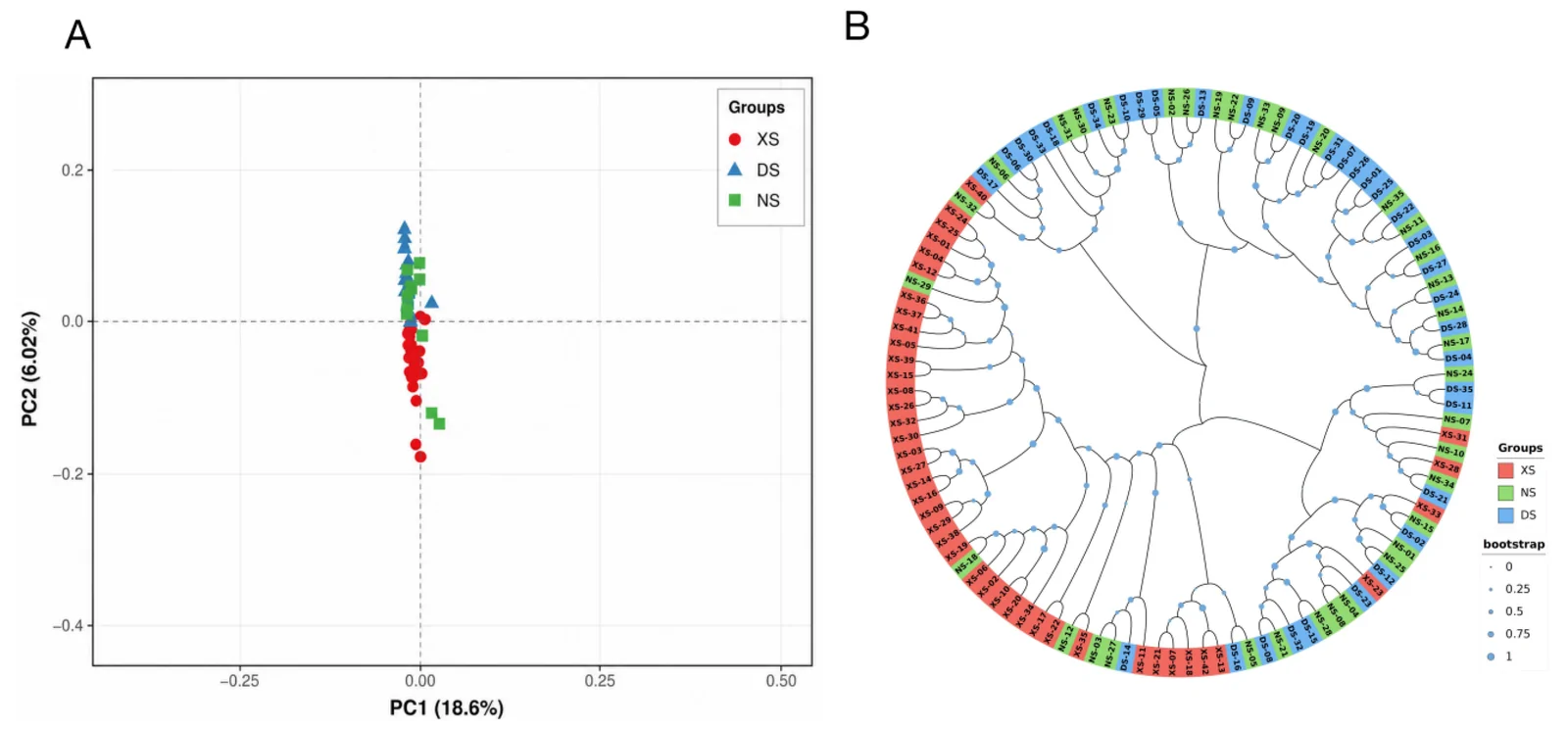
Population genetic structure analysis of *D. macarellus* based on genomic SNP data. (**A**) Principal component analysis plot illustrating genetic similarity among individuals from three geographic groups. (**B**) Phylogenetic tree depicting phylogenetic relationships among individuals.

**Figure 5:**
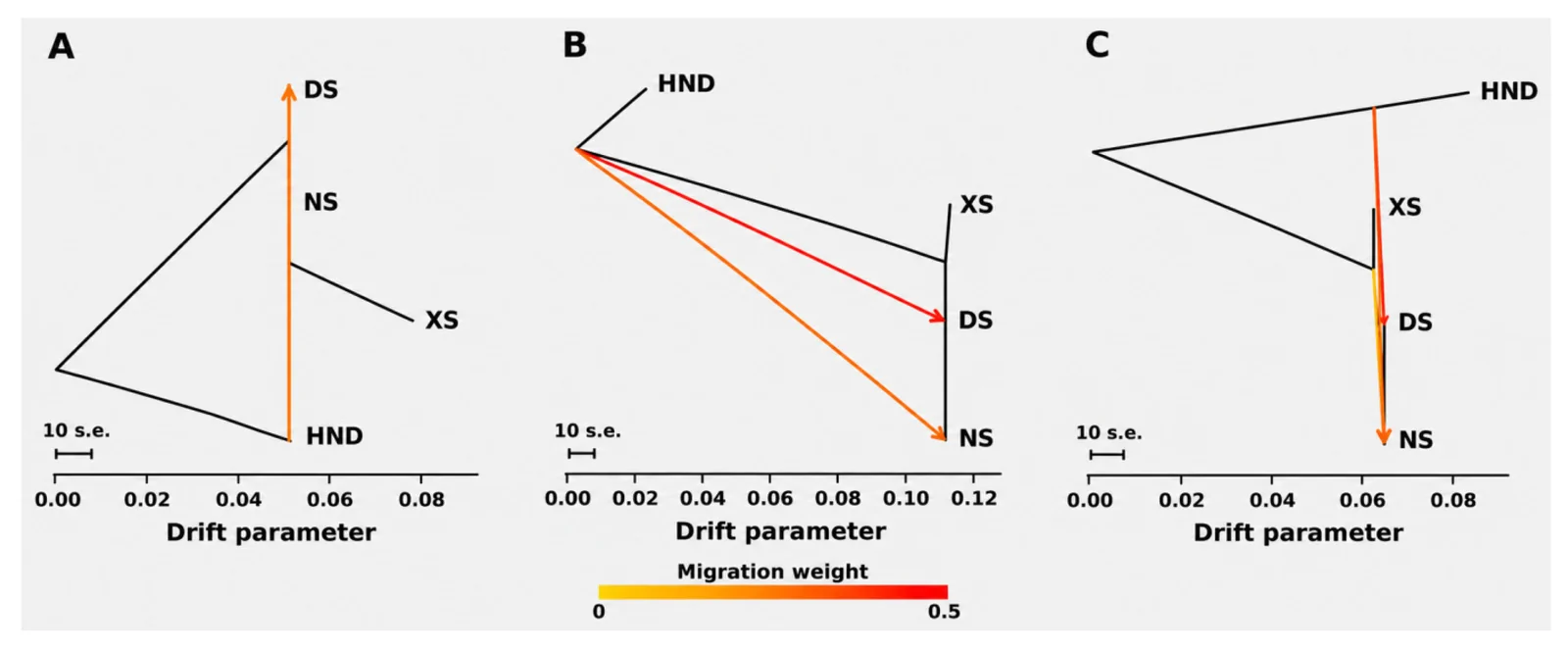
TreeMix analysis of historical relationships and migration-edge signals among *D*. *macarellus* sampling groups. (**A**–**C**) TreeMix models with one, two, and three migration edges (*m* = 1–3), respectively.

**Figure 6:**
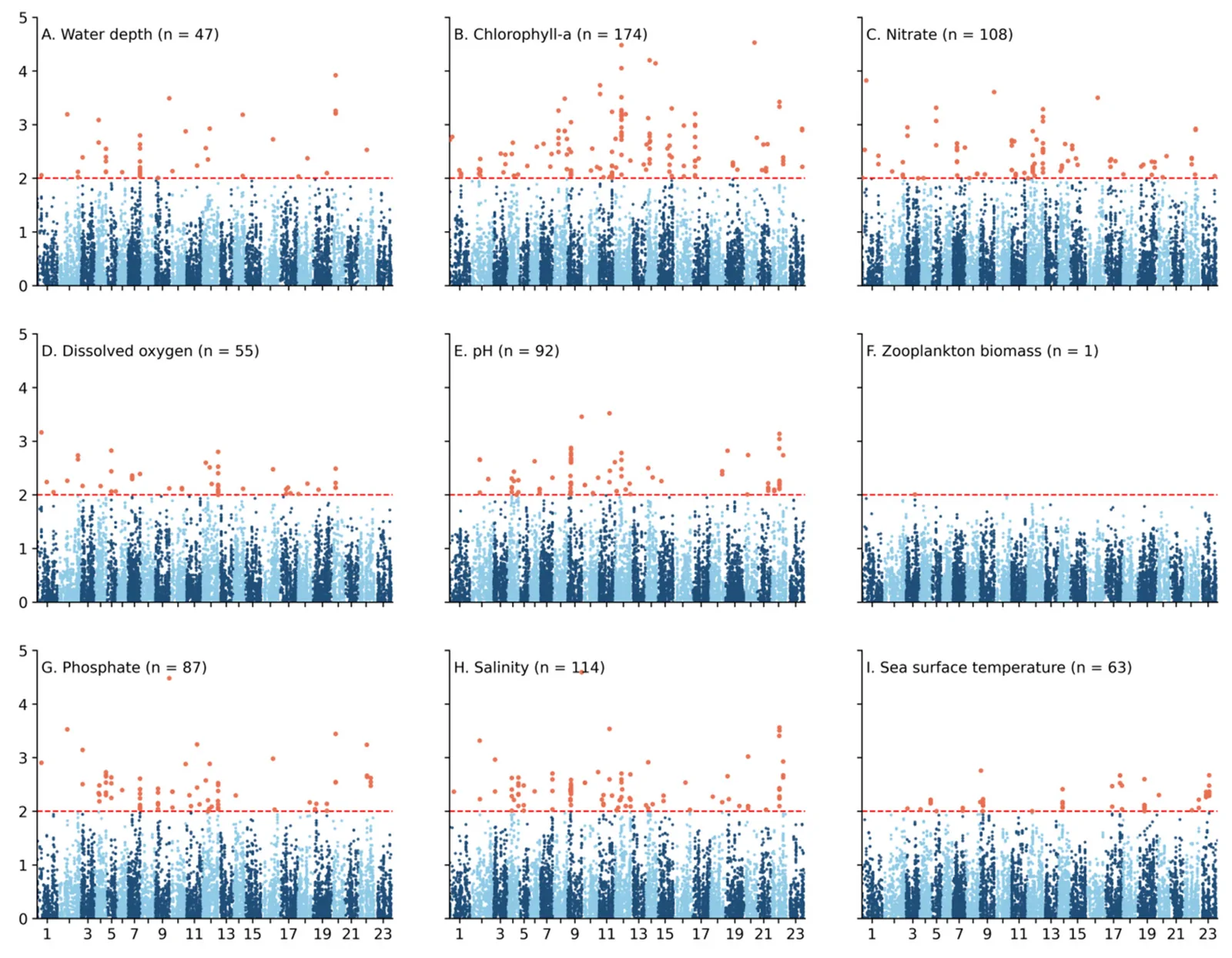
Manhattan plots of genotype–environment associations in *D*. *macarellus*. (**A**) Water depth; (**B**) chlorophyll-a; (**C**) nitrate; (**D**) dissolved oxygen; (**E**) pH; (**F**) zooplankton biomass; (**G**) phosphate; (**H**) salinity; and (**I**) sea surface temperature. Candidate SNPs with corrected *p* < 0.01 are highlighted in red, and the red dashed line indicates the candidate threshold of corrected *p* = 0.01. The alternating dark- and light-blue points distinguish SNPs on different chromosomes.

**Table 1:** Genetic diversity indices for three geographic sampling groups of *Decapterus macarellus.*

Sampling Sites	*H*_O_	*H*_E_	MAF	π
XS	0.0249695	0.0314444	0.0167241	0.031914
DS	0.0166909	0.0200374	0.0106122	0.020452
NS	0.0171157	0.0205026	0.0106779	0.020887

**Table 2:** Pairwise fixation indices (F_ST_) and genetic distances (D_XY_) among three geographic sampling groups of *Decapterus macarellus.*

	NS	DS	XS
NS		0.0086	0.0102
DS	0.0016		0.0120
XS	0.0017	0.0017	

Note: Above the diagonal represents pairwise fixation indices (F_ST_); below the diagonal represents genetic distances (D_XY_).

## Data Availability

The raw sequencing reads generated in this study are available in the NCBI Sequence Read Archive (SRA) under BioProject accession number PRJNA1505054.
